# Significant contribution of stacking faults to the strain hardening behavior of Cu-15%Al alloy with different grain sizes

**DOI:** 10.1038/srep16707

**Published:** 2015-11-19

**Authors:** Y. Z. Tian, L. J. Zhao, S. Chen, A. Shibata, Z. F. Zhang, N. Tsuji

**Affiliations:** 1 Shenyang National Laboratory for Materials Science, Institute of Metal Research, Chinese Academy of Sciences, 72 Wenhua Road, Shenyang 110016, P.R. China; 2 Department of Materials Science and Engineering, Kyoto University, Yoshida-honmachi, Sakyo-ku, Kyoto 606-8501, Japan; 3 Elements Strategy Initiative for Structural Materials (ESISM), Kyoto University, Yoshida-honmachi, Sakyo-ku, Kyoto, 606-8501, Japan

## Abstract

It is commonly accepted that twinning can induce an increase of strain-hardening rate during the tensile process of face-centered cubic (FCC) metals and alloys with low stacking fault energy (SFE). In this study, we explored the grain size effect on the strain-hardening behavior of a Cu-15 at.%Al alloy with low SFE. Instead of twinning, we detected a significant contribution of stacking faults (SFs) irrespective of the grain size even in the initial stage of tensile process. In contrast, twinning was more sensitive to the grain size, and the onset of deformation twins might be postponed to a higher strain with increasing the grain size. In the Cu-15 at.%Al alloy with a mean grain size of 47 μm, there was a stage where the strain-hardening rate increases with strain, and this was mainly induced by the SFs instead of twinning. Thus in parallel with the TWIP effect, we proposed that SFs also contribute significantly to the plasticity of FCC alloys with low SFE.

Materials with high strength and high ductility are widely attractive for industrial applications^1^. In general, the yield strength of materials is determined by the grain size according to the Hall-Petch relation^2^, and the ductility (especially uniform elongation) is governed by the strain hardening rate (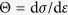, where 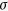 and 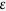 are true stress and true strain, respectively) according to plastic instability condition, such as Considére criterion^2^. However, it is well known that strength and ductility have trade-off relationship in many metallic materials^3^,^4^. Nevertheless, there are some exceptions. One type of these materials contain high density nano-sized growth twins or deformation twins^5^,^6^,^7^,^8^,^9^. In these cases, twins have a remarkable effect on the mechanical properties of face-centered cubic (FCC) alloys. Recent studies show that the FCC alloys with low stacking fault energy (SFE) always exhibit high strain hardening rate and good balance of strength and ductility, where deformation twinning is supposed to play a key role^8^,^10^,^11^,^12^,^13^,^14^. In these materials, the propensity of generating perfect dislocations, stacking faults (SFs) or/and twins must affect the strain-hardening behavior thus determining the strength and ductility. To make a better understanding on the origin of superior mechanical properties of materials with low SFE, it is significantly important to investigate their strain-hardening behaviors.

In the previous studies, the strain-hardening behaviors of FCC metals and alloys with high SFE have been studied extensively and the substructure configurations at different stages of plastic deformation have been clarified^15^. For the low-SFE materials, their strain-hardening behaviors are different due to formation of deformation twins and planar dislocations^16^,^17^,^18^. For example, the strain-hardening rate of single-phase Cu-Al alloys is generally higher than that of pure Cu since the SFE of Cu-Al alloys is much lower than pure Cu^18^. In addition, there is always a deformation stage where the strain-hardening rate increases with strain in coarse-grained (CG) materials with low SFE^16^,^17^,^18^. Gutierrez-Urrutia *et al.*^16^ have systematically studied the strain-hardening behavior and substructure evolution in a Fe-22 wt.%Mn-0.6 wt.%C (hereafter Fe-22Mn-0.6C) austenitic steel with a mean grain size of 50 μm, and they observed an increased strain-hardening stage which was attributed to the deformation twinning. This kind of steels are also famous as twinning-induced plasticity (TWIP) steels. Similarly, a stage of increased strain-hardening rate was also detected in a 70/30 brass with mean grain sizes of 30 μm and 250 μm, respectively, and it was also suggested that this was induced by deformation twins^17^. However, the substructure evolution during tensile test was not fully investigated yet in these Cu alloys with low SFE^19^. In comparison with these reports where twinning effect is emphasized, Meyers *et al.*^20^ reported that SFs, instead of twins, may dominate the deformation behavior in single crystalline Cu-Al alloys in laser shock compression experiments. It is thus desirable to clarify the roles played by SFs and twins in FCC materials with low SFE.

For the FCC metals and alloys with low SFE, the grain size also plays a crucial role in determining the deformation mechanisms^17^,^21^,^22^,^23^. In coarse-grain regime, it was empirically reported that smaller grain size impeded deformation twinning^22^,^23^. El-Danaf *et al.*^17^ investigated the effect of grain size on the mechanical behavior of a low-SFE 70/30 brass having different grain sizes (9 μm, 30 μm and 250 μm), and they found that the strain-hardening curves were significantly different. However, further studies are required to clarify the microstructure evolution and related deformation mechanisms. It is valuable to note that the grain sizes always fall in the CG regime, for example large than 1 μm in the previous studies. In contrast, we have fabricated fully recrystallized ultrafine-grained Fe-22Mn-0.6C austenitic steel^24^ and Cu-15 at.%Al (hereafter Cu-15Al) alloy^12^, both with low SFEs, by conventional cold rolling and annealing processes. The minimum mean grain sizes achieved were about 550 nm for the Fe-22Mn-0.6C steel^24^ and about 600 nm for the Cu-15Al alloy^12^. This makes it possible to investigate the strain-hardening behavior of low-SFE materials from the CG regime to the submicronmeter regime systematically. The current study is thus initiated to reveal the effect of grain size on the strain-hardening behavior and to clarify the contribution of either perfect dislocations, SFs or/and twins during tensile deformation in a Cu-15Al alloy with low SFE.

## Results

**Figure 1:**
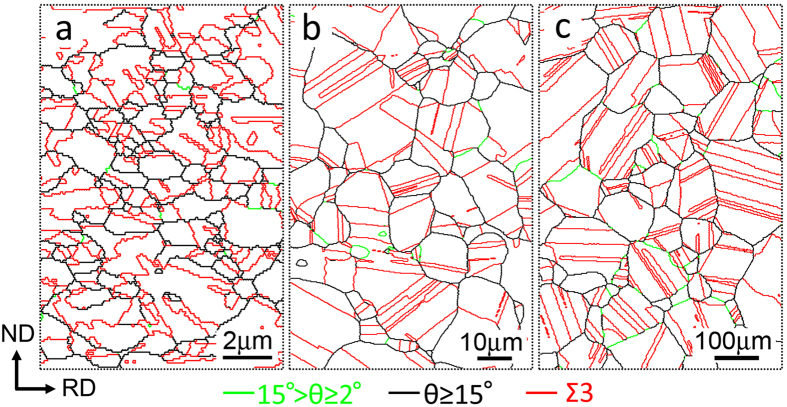
EBSD maps of the Cu-15Al specimens with different grain sizes. (**a**) FG specimen; (**b**) MG specimen; (**c**) CG specimen. The green, black and red lines are related to low-angle grain boundaries, high-angle grain boundaries and twin boundaries, respectively.

Figure 1 shows the EBSD grain boundary images of the samples after annealing following to cold-rolling. The green, black and red lines are related to low-angle grain boundaries (LAGBs), high-angle grain boundaries (HAGBs) and twin boundaries (TBs), respectively. All the samples were fully recrystallized, and the mean grain sizes were measured to be 0.6 μm, 7 μm and 47 μm for Fig. 1a–c, respectively. Note that all the HAGBs including TBs were counted for the measurement of the mean grain sizes. For simplicity, the three kinds of materials are referred as fine-grained (FG) specimen, medium-grained (MG) specimen and coarse-grained (CG) specimen, respectively.

### Grain size effect on the mechanical properties

**Figure 2:**
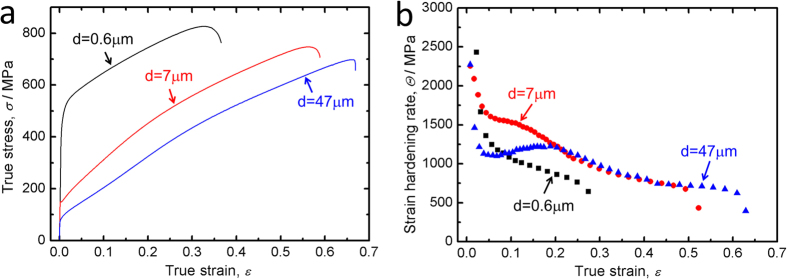
Mechanical properties of the Cu-15Al specimens with different grain sizes. (**a**) True stress-strain curves; (**b**) strain-hardening curves.

Figure 2 shows the true stress-strain curves and strain-hardening curves of the Cu-15Al alloy samples with different grain sizes. With decreasing the grain size from 47 μm to 0.6 μm, the yield strength greatly increases from 80 MPa to 450 MPa, whereas the uniform elongation decreases from 82% to 28%, as shown in Fig. 2a. It is noted that the elongation especially the uniform elongation is fairly large even if the grain size is only 0.6 μm. Meanwhile, the effect of grain size on the strain-hardening behavior was also checked. Fig. 2b shows the strain-hardening rates plotted as a function of true strains for the three kinds of samples. It was found that the three strain-hardening curves diverse significantly. Each curve was analyzed and deformation patterns at specified strains were carefully characterized to clarify the fundamental mechanisms.

### Microstructures after tensile testing to different strains

**Figure 3:**
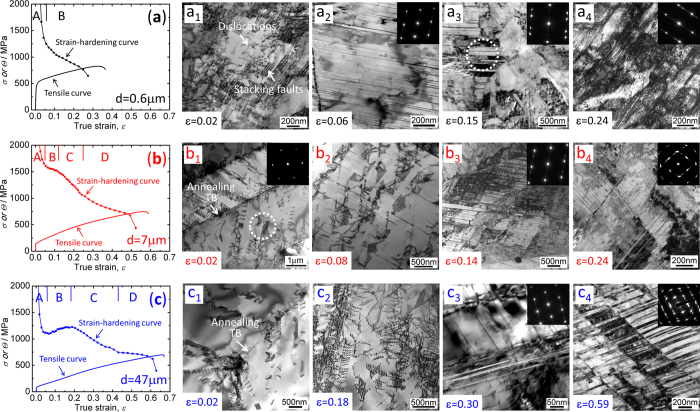
Strain-hardening curves, tensile stress-strain curves and typical TEM images at specific strains. (**a–c**) True stress-strain curve and strain-hardening curve of the FG specimen, MG specimen and CG specimen, respectively, where (**A–D**) are corresponding to the strain-hardening stages; (a_1_–a_4_) typical TEM images of the FG specimen developed at specific strains during tensile test; (b_1_–b_4_) typical TEM images of the MG specimen developed at specific strains during tensile test; (c_1_–c_4_) typical TEM images of the CG specimen developed at specific strains during tensile test. Y-axis of Fig. 3a–c is related to the true stress (σ) or strain-hardening rate (Θ). All the photographs were taken by Y.Z. Tian.

Figure 3 shows the strain-hardening curves, tensile stress-strain curves and related deformation patterns (TEM images) at specified strains. For the FG specimen, two stages of strain hardening can be recognized, as shown in Fig. 3a. It is apparent that the strain-hardening rate decreases sharply in stage A (ε < 0.06), and then it decreases steadily in stage B (ε > 0.06). For the MG specimen, the strain hardening curve could be divided into four stages in Fig. 3b, which were defined as A (ε < 0.05), B (0.05 < ε < 0.12), C (0.12 < ε < 0.25) and D (ε > 0.25), respectively. In stage A, the strain-hardening rate decreases significantly, which is similar to the stage A in the FG specimen. After that, a plateau appears in stage B. It is suggested that this plateau is related to the onset of primary twinning previously observed during compression of a Cu-30 wt.%Zn alloy^17^,^25^. With increasing the strain, the strain-hardening rate decreases in stages C and D. For the CG specimen, four stages were also found in Fig. 3c, which were defined as A (ε < 0.06), B (0.06 < ε < 0.18), C (0.18 < ε < 0.43) and D (ε > 0.43), respectively. The strain hardening curve of the CG specimen is similar to the MG specimen except in stage B, where the strain-hardening rate increases with increasing the true strain. Similar stages were also found in a Cu-13Al alloy with a grain size of 50 μm^18^ and a Fe-22Mn-0.6C TWIP steel with a grain size of 50 μm^16^.

Microstructures were then characterized at specified strains to clarify the inherent deformation mechanisms related to the strain-hardening behavior. For the FG specimen, a large number of SFs but a limited number of dislocations were found generally when the tensile strain was very small (0.02), as shown in Fig. 3a_1_. With increasing the tensile strain to 0.06, some deformation twins emerged, which were confirmed by the selected area electron diffraction (SAED) pattern shown in Fig. 3a_2_. However, SFs still dominated at this strain level. At a strain of 0.15, deformation twins began to increase remarkably. Fig. 3a_3_ exhibits a typical deformation twin pattern at this strain level, where the SAED pattern is taken from the circle region. Fig. 3a_4_ displays a TEM image of the specimen tensioned to a strain of 0.24 at which macroscopic necking starts. Numerous SFs and deformation twins appear, as revealed from the inserted SAED pattern. The microstructural evolution delineated above indicates that SFs and dislocations dominate in stage A, while in contrast deformation twinning begins to play an important role in stage B in the FG specimen.

For the MG specimen, the microstructural evolution during tensile test is significantly different in contrast to the FG specimen. Fig. 3b_1_ shows the TEM image at a true strain of 0.02. The straight lines are annealing TBs, as indicated by the arrows. It was found that numerous dislocations pile up in front of the annealing TBs; besides, there are also some SFs as enclosed in the circle, where the SAED pattern is taken from the same region. It should be noted that dislocation slip dominates the plastic deformation at this small strain level, which is different from the FG specimen. With increasing the strain level to ε = 0.08, SFs were detected more frequently in addition to dislocations as shown in Fig. 3b_2_. This indicates that the deformation mechanisms in stages A and B have somehow transferred. However, no deformation twin was detected. At a strain of ε = 0.14, SFs again prevailed and dislocations could also be distinguished. In addition, deformation twins emerged as displayed in Fig. 3b_3_, which were confirmed by the inserted SAED pattern. When the tensile strain was increased to 0.24, Fig. 3b_4_ shows SFs and deformation twins which intersect mainly along two directions. Besides, it is very difficult to distinguish individual dislocation at such a high strain level. The microstructural evolution mentioned above indicates that SFs and dislocations dominate in stages A and B; in contrast, deformation twinning begins to play a role in stage C in the MG specimen.

For the CG specimen, the microstructural evolution during tensile test is similar to the MG specimen. At a true strain of 0.02, numerous dislocations and limited SFs were found, as shown in Fig. 3c_1_, where the annealing TB was indicated by an arrow. This is similar to the microstructure of the MG specimen at the same strain level as shown in Fig. 3b_1_, but the dislocation density is lower in this CG specimen. With increasing the strain, the densities of dislocations and SFs increase remarkably. At a true strain of ε = 0.18, both dislocations and SFs were detected again, as shown in Fig. 3c_2_. These results indicated that the dominating deformation patterns were dislocations and SFs in stage B, but no deformation twin was observed although the strain-hardening rate dramatically increases. When the true strain is 0.3, only limited deformation twins were detected, as confirmed by the SAED pattern in Fig. 3c_3_. However, the dominating deformation patterns are still SFs and dislocations. At a true strain of ε = 0.59 which related to the necking strain, numerous SFs and deformation twins were detected, as revealed from the SAED pattern shown in Fig. 3c_4_. At this high strain level, dislocation accumulation in the form of banded structure was detected due to the limited recovery process. The microstructural evolution process delineated above indicates that SFs and dislocations dominate in stages A and B; in contrast, deformation twinning begins to play a role in stage C in the CG specimen. Above all, the microstructural evolution of the CG specimen after tensile testing is similar to the MG specimen, except that the emergence of deformation twins is postponed to a higher strain level with increasing the grain size.

## Discussion

### Features of grain size effect on the tensile deformation mechanisms

**Figure 4:**
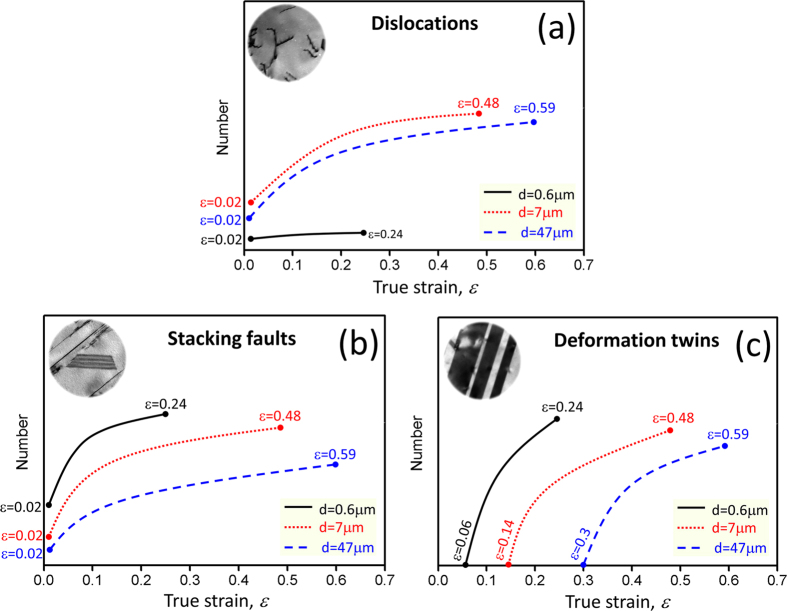
Schematic illustration on the deformation patterns developed with increasing the tensile true strain. (**a**) Dislocations; (**b**) stacking faults and (**c**) deformation twins in the Cu-15Al alloy with different grain sizes. Dislocations and stacking faults were detected at the small strain of 0.02; deformation twins emerge at different strains of 0.06, 0.14 and 0.3 for the FG, MG and CG specimens, respectively. The numbers of dislocations, stacking faults and deformation twins were statistically estimated with increasing the strain until necking. The strains of 0.24, 0.48 and 0.59 are corresponding to the strains at necking points for the FG, MG and CG specimens, respectively. X-axis is related to the tensile true strain.

For FCC metals and alloys with low SFE, SFs and deformation twins are favored in contrast to those materials with high SFE under plastic deformation^11^,^26^. In the present study, systematic results indicate that grain size influences significantly on the deformation mechanisms and the structure configurations of a Cu-15Al alloy during tensile test. Fig. 4 schematically shows the evolution of dislocations, SFs and deformation twins with tensile strain in the three Cu-15Al specimens with different grain sizes. Dislocations were detected from the very small strain of 0.02 until necking in the three samples, as shown in Fig. 4a. In the FG specimen, the planar dislocation activity was quite limited at the strain of 0.02 and rapid increase of the dislocation density was not observed. In the MG and CG specimens, however, dislocations were highly developed at the strain of 0.02 and multiplied rapidly with increasing the strain. For the SFs, they were limited in the MG and CG specimens but appeared predominantly in the FG specimen at the strain of 0.02, which is the opposite tendency to the dislocations. With increasing the strain, SFs multiplied significantly in the three samples, as shown in Fig. 4b. For the deformation twins, it was interesting to note that they emerged at different true strains of 0.06, 0.14 and 0.3 for the FG, MG and CG specimens, respectively. With increasing the strain, deformation twins increased significantly in the three samples, as shown in Fig. 4c. These results indicate that with increasing the grain size, dislocation slip was highly activated, but the onset of deformation twinning was postponed to a higher strain level. In contrast, SFs existed throughout the tensile process, indicating that SFs may contribute significantly to the plastic deformation of the Cu-15Al alloys.

The above-mentioned microstructural evolution process indicates that grain size affects significantly on dislocation slip, SFs and twinning activity in the Cu-15Al alloy. It has been reported that grain refinement will make twinning difficult in the coarse-grained regime^22^,^23^. Recent studies indicate that crystallographic orientation, SFE and grain size are all key parameters in determining the deformation twinning behavior of FCC materials^27^. With decreasing the grain size, dislocation slip would be increasingly inhibited and the activation of deformation twins became relatively easier at a critical grain size^27^,^28^. Concerning the critical strains for twinning in the present study, the related twinning stress of the FG specimen is ~600 MPa, which is higher than ~400 MPa of the MG and CG specimens. Present results coincide with the above mentioned predictions^22^,^23^,^27^,^28^. For the SFs, they appeared at a small strain of 0.02 and increased rapidly with increasing the strain, indicating that SFs can play an important role in sustaining the plasticity of the Cu-15Al alloy. In fact, recent report by Meyers *et al.*^20^ suggests that SFs, instead of twinning, dominate in the deformation behavior during laser shock compression of single crystalline Cu-Al alloys with different Al contents. The present paper clearly shows the importance of SFs on deformation in conventional tensile test of a polycrystalline material.

In accordance with the deformation patterns, the strain-hardening behavior is also different for the three specimens. When the strain is smaller than 0.03, the strain-hardening rate of the FG specimen is higher than the MG and CG specimens, as shown in Fig. 2b. This is possibly because SFs dominate in the FG specimen but dislocations dominate in the MG and CG specimens at this early stage, and SFs can enhance strength much higher than dislocations. However, the strain-hardening rate decreases continuously in the FG specimen since it becomes increasingly difficult to introduce more SFs or other deformation mechanisms within small grains (small spaces) at higher strains. In comparison with the FG specimen, the strain-hardening rates of the MG and CG specimens are much higher when the strain is larger than 0.08, and this may be attributed to the multiple strengthening mechanisms involved during the tensile deformation. In that case, the strain-hardening behaviors of the MG and CG specimens should be compared.

In contrast to the CG specimen, the strain-hardening rate is much higher for the MG specimen, as shown in Fig. 2b, when the strain is smaller than 0.2. This is because, for the MG specimen, the densities of dislocations and SFs are higher at the early stage of tensile straining in contrast to the CG specimen, as exhibited in Fig. 3b_1_,c_1_. In addition, deformation twins were also recognized when the strain approached to 0.14 for the MG specimen whereas no deformation twins were detected in the CG specimen when the strain was smaller than 0.2. Overall, the higher densities of dislocations and SFs and the early onset of deformation twinning are responsible for the higher strain-hardening rate for the MG specimen in comparison with the CG specimen. When the strain is larger than 0.2, the strain-hardening curves of the MG and CG specimens matched very well. Note that if the strain-hardening curve of the FG specimen was shifted to the higher strain region in Fig. 2b, stage B of the FG specimen can also match with the MG and CG specimens. This is possibly because the deformation mechanisms are originated mainly from SFs and deformation twinning in the later stages of strain-hardening for the three specimens.

### Significant roles of SFs

In FCC metals and alloys with low SFE, deformation twins were frequently observed during plastic deformation^11^,^29^,^30^,^31^. In contrast to dislocation accumulation, TB can strengthen the materials more efficiently as TB is a perfectly coherent HAGB and the twin/matrix thickness always falls into the nanoscale. Thus TB can impede the dislocation movement efficiently, inducing a significant increase in strength. For example, Meyers *et al.*^23^ summarized the Hall–Petch slopes for both perfect dislocation slip and twinning in a number of materials with FCC, body-centered cubic (BCC) and hexagonal close-packed (HCP) crystal structures. In each case, the experimentally observed Hall–Petch slope for twinning is higher than that for the dislocation slip^23^. As a result, for those materials with low SFE where deformation twinning involves, there is always a higher strain-hardening rate. For example, in CG Fe-22Mn-0.6C austenitic steel with a grain size of 50 μm, there is a stage where the strain-hardening rate increases significantly with strain during tensile test, and it is proposed that this stage is related to the onset of deformation twinning^16^. In a low-SFE Cu-30Zn alloy with a grain size of 25 μm, there is a plateau stage, which is also suggested to be induced by deformation twinning^25^. In the present study, a plateau region in the MG specimen and an increased strain-hardening stage in the CG specimen appeared but different deformation patterns were obtained concerning the role of deformation twinning during tensile test. For the FG specimen, deformation twins were observed when the tensile strain is only 0.06, and they play an important role in sustaining the strain hardening rate. For the MG specimen, deformation twins did not appear until the strain approached to 0.14, which exceeds the strain range of stage B, as illustrated in Fig. 3b. This indicates that the plateau stage B may be not induced by deformation twins. Similarly, for the CG specimen, deformation twins did not appear until the strain of 0.3, thus the increase of strain-hardening rate in stage B should be not induced by deformation twins. Previous study indicates that dislocation glide dominates the deformation of pure Cu during tensile test at room temperature, and the strain-hardening rate decreases monotonously with increasing the strain irrespective of the grain size^32^. Concerning the large number of SFs besides dislocations, they may play a key role in inducing the plateau region B in the MG specimen and the region B in the CG specimen where strain-hardening rate increases with strain.

Recent theoretical studies by molecular dynamics simulation reveal the interaction between dislocations and SFs, and it is found that in most cases SFs prevent the glide of other dislocations on slip planes crossing the SF plane^33^,^34^. This supports the present experimental fact that SFs play an important role in enhancing strain hardening, leading to the plateau region of the MG specimen and the increased strain-hardening region in the CG specimen. Overall, the present results have clearly provided a new deformation mechanism in low-SFE materials during tensile test. For the Cu-15Al alloy, deformation twinning indeed plays an important role in sustaining the plasticity but only at the later stage during the tensile test. In contrast, SFs appear just after yielding and they are increasingly activated with increasing the tensile strain until fracture, indicating that SFs may be even more crucial in sustaining the ductility in the Cu-Al alloy with low SFE.

### Strain-hardening behaviors of materials with different SFEs

**Figure 5:**
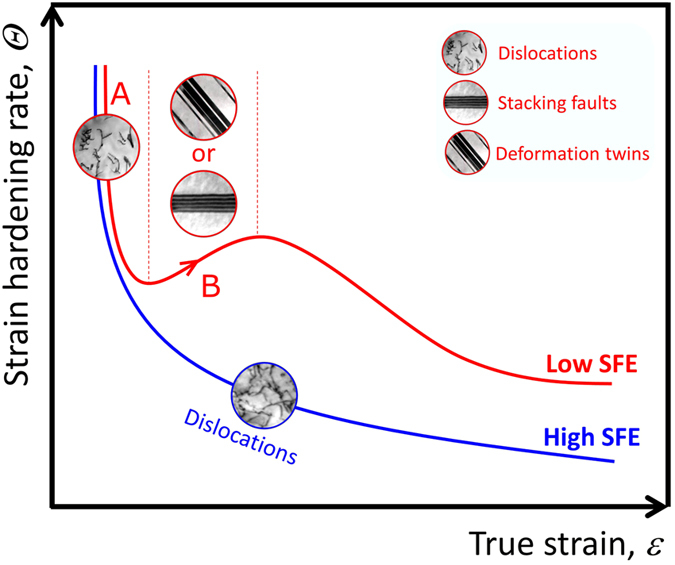
Schematic illustration on the typical strain-hardening curves of the coarse-grained FCC materials with high and low SFEs. (**A**,**B**) are related to two typical strain-hardening stages of FCC materials with low SFE. Deformation patterns of dislocations, stacking faults and deformation twins were inserted at different stages.

In the material with a high SFE, dynamic recovery is favored due to the dislocation cross slip under the quasi-static straining process^11^,^35^. In that case, the strain-hardening rate is monotonously decreased with increasing the strain^32^. In contrast, the strain-hardening behavior is sensitive to the grain size in the material with a low SFE due to the intervening of SFs and deformation twins, as delineated above. Fig. 5 schematically shows the strain-hardening curves of CG FCC materials with low and high SFEs. In the CG alloy with low SFE, multiple stages were described from the strain-hardening curve. In the stage A, dislocations dominate the plastic deformation during the straining process though SFs also appear, thus the strain-hardening rate decreases sharply in this stage, which is similar to the material with a high SFE. Further increase in strain will activate more SFs and dislocations, which will affect the strain-hardening rate significantly. It is noteworthy that in the CG alloy, there is a stage B where the increase of strength is accelerated with increasing the strain in the form of an enhancement of strain-hardening rate. In the previous study, it has been considered that this stage is induced by the activity of deformation twinning, and this phenomenon is frequently observed in high-Mn TWIP steels^16^. Since the SFE of the Cu-15Al alloy is quite low, it is expected that similar TWIP effect would contribute significantly to the stage B^6^. However, SFs instead of deformation twins were detected in this stage. It is thus concluded that both SFs and deformation twins can induce the increase of strain hardening rate. Though both Cu-Al alloy and Fe-22Mn-0.6C steel are FCC materials with low SFE, it seems that the activation of deformation twins are quite different which may be owing to the solid solution type, i.e., interstitial C atoms may play a key role in activating deformation twins at earlier stage in the Fe-22Mn-0.6C steel.

In summary, we compared the grain size effect (0.6 μm, 7 μm, 47 μm) on the strain-hardening behavior and deformation mechanisms of fully recrystallized Cu-15Al alloys with low SFE. It is well accepted in the previous study that deformation twinning can block dislocations efficiently and enhance the strain-hardening rate significantly, postponing the necking process thus improving the strength and ductility of materials with low SFE, which is known as the general “TWIP effect”^6^,^16^. In this study, however, we clearly demonstrated a new deformation mechanism that was SF-induced plasticity during tensile process. There existed a stage where the strain-hardening rate increased with strain in the CG Cu-15Al alloy, where dislocations and SFs were observed but no deformation twin was found. It is thus proposed that the enhancement of strain-hardening rate in this stage is mainly induced by SFs instead of deformation twins. We also evaluated the roles played by dislocation slip, SFs and deformation twins in the three Cu-15Al alloy samples with different grain sizes. Dislocations and SFs appeared throughout the tensile process irrespective of the grain size, and a difference laid in the density in the initial straining stage. Thus SFs can contribute significantly to the plasticity of Cu-Al alloys with low SFE. For the deformation twinning, it was sensitive to the grain size, and the onset of twinning was postponed to a higher strain with increasing the grain size, indicating that deformation twinning played a key role in the later straining stage during tensile process.

## Methods

### Materials fabrication

The starting material was a Cu-14.66 at.%Al (defined as Cu-15Al for simplicity) alloy rod with 25.4 mm in diameter, and the mean grain size was about 67 μm. The SFE of the Cu-15Al alloy is about 7 mJ/m^2^ according to the reported data in the literatures^11^,^18^,^36^,^37^. The rod was firstly cold rolled to a reduction in thickness of 96%. Specimens with different grain sizes of 0.6 μm (FG specimen), 7 μm (MG specimen) and 47 μm (CG specimen) were prepared by subsequent isothermal annealing at 400 °C, 550 °C and 800 °C for 40 min, 2 h and 2 h, respectively.

### Tensile test

Tensile tests were conducted at an initial strain rate of 8.3 × 10^−4^ s^−1^ using a Shimadzu tensile testing machine at ambient temperature. Tensile specimens with gauge length of 10 mm, width of 5 mm and thickness of 1 mm were cut from the sheets by electrical discharge machine. The strains in the tensile test were accurately measured by an extensometer until necking. Some specimens were also tensioned to specific strains (0.02, 0.06, 0.15 and 0.24 in the FG specimen, 0.02, 0.08, 0.14, 0.24 and 0.48 in the MG specimen, 0.02, 0.08, 0.18, 0.3 and 0.59 in the CG specimen) and unloaded to characterize the corresponding deformation substructures.

### Microstructure characterization

After annealing, microstructure characterizations were undertaken by using a field-emission scanning electron microscopy (FE-SEM, FEI (Philips) Siron) equipped with an electron backscattering diffraction (EBSD) system operated at an accelerating voltage of 15 kV. For the EBSD images, sections perpendicular to the transverse direction (TD) of the sheets were characterized. EBSD samples were firstly ground using 4000 grit SiC polishing paper and then electro-polished in a solution of 25% H_3_PO_4_, 25% C_2_H_5_OH and 50% deionized water with a voltage of 10 V at about 0 °C. During the EBSD measurement, different step sizes of 100 nm, 250 nm and 2.5 μm were used to characterize the recrystallized microstructure of the FG, MG and CG specimens, respectively. After the EBSD characterization, grain sizes of the recrystallized samples were measured manually by the linear intercept method by counting all the high-angle grain boundaries (HAGBs) including twin boundaries (TBs).

Transmission electron microscopy (TEM) characterization was conducted using a JEOL 2010 machine operated at 200 kV. TEM samples were cut from the surface of the tensile specimens which were tensioned to different true strains. The observation plane of the TEM specimen is parallel to the surface of the tensile specimen. They were firstly ground to 60 μm using 4000 grit SiC polishing paper and then twin-jet electro-polished by Struers Tenupole-3 in a solution of 25% H_3_PO_4_, 25% C_2_H_5_OH and 50% deionized water with a voltage of 10 V at ambient temperature.

## Additional Information

**How to cite this article**: Tian, Y. Z. *et al.* Significant contribution of stacking faults to the strain hardening behavior of Cu-15%Al alloy with different grain sizes. *Sci. Rep.* **5**, 16707; doi: 10.1038/srep16707 (2015).
